# Psychometric evaluation of the Sinhalese version of MacNew Heart Disease Health Related Quality of Life Questionnaire in patients with stable angina

**DOI:** 10.1186/s12955-016-0448-0

**Published:** 2016-03-15

**Authors:** K. L. M. D. Seneviwickrama, D. B. D. L. Samaranayake, P. Fonseka, G. N. L. Galappaththy, S. Höfer, N. B. Oldridge

**Affiliations:** Nutrition Coordination Division, 555/5 Elvitigala Mawatha, Colombo 5, Sri Lanka; Department of Community Medicine, University of Colombo, Colombo, Sri Lanka; Department of Community Medicine, University of Sri Jayawardhenepura, Nugegoda, Sri Lanka; Emergency Response to Artemisinin Resistance (ERAR) and other Vector Borne & Parasitic Diseases, World Health Organization, Hà Nội, Viet Nam; Department of Medical Psychology, Innsbruck Medical University, Innsbruck, Austria; College of Health Sciences, University of Wisconsin-Milwaukee, Milwaukee, USA

**Keywords:** Factor analysis, Health-related quality of life, MacNew, Stable angina, Validation

## Abstract

**Background:**

A Sinhalese version of a validated, disease-specific patient-reported heart disease health related quality of life instrument is lacking. The purpose of this study was to validate the interviewer-administered Sinhalese version of the MacNew Heart Disease Health-related Quality of Life Questionnaire (MacNew) in patients with clinically diagnosed stable angina.

**Methods:**

The Sinhalese translation of the MacNew was carried using standard forward- backward translation technique. In this validation study, the MacNew was administered to 200 patients with stable angina. Reliability was assessed by internal consistency and test-retest reliability. Construct validity was explored by exploratory factor analysis using principal component analysis and confirmed by confirmatory factor analysis using the robust maximum likelihood method and known group comparison. The correlation between compatible domain scores of MacNew and the World Health Organization’s quality of life –brief questionnaire was used to assess concurrent validity.

**Results:**

The original 3-factor model (Physical, Emotional and Social) of the MacNew with cross-loadings was confirmed: principal component analysis with 53.42 % of the explained variance and confirmatory factor analysis with adequate fit for each of the three model fit criteria considered [root mean square error of approximation = 0.044 (90 % CI = 0.031 to 0.056); comparative fit index = 0.99; χ^2^/df = 1.39]. Internal consistency of the MacNew was acceptable with Cronbach's α of 0.92 on the Global scale and on the domain scales ranging from 0.85–0.91. Test-retest reliability was also found to be satisfactory with intraclass correlation coefficients of >0.9 for total and domain scores. A satisfactory level of concurrent validity was demonstrated with statistically significant correlations between compatible domain scores of MacNew and the World Health Organization’s quality of life questionnaire (Pearson correlation ranging from 0.36−0.79).

**Conclusions:**

The interviewer–administered Sinhalese MacNew is a valid and reliable patient-reported outcome measure to assess disease specific health-related quality of life among Sinhalese patients with stable angina.

## Background

Clinicians and policy makers increasingly recognize the importance of measuring health-related quality of life (HRQL) to inform patient management and policy decisions and it has become an important outcome measure in the evaluation of health interventions in chronic diseases including cardiology [1, 2]. Two basic approaches to HRQL measurement are available: generic instruments that are applicable across a wide range of populations and specific instruments that focus on problems associated with single disease states, patient groups, or areas of function [3, 4].

The MacNew HRQL questionnaire (MacNew) is one of the more widely used coronary artery disease (CAD) -specific HRQL questionnaires [5]. The MacNew is a self-administered modification [6] of the original interviewer-administered Quality of Life after Myocardial Infarction questionnaire [7] and has since been translated into over 40 languages and psychometrically validated in 22 languages in patients with myocardial infarction, angina and ischaemic heart failure [8]. The MacNew consists of 27 questions with a Global HRQL score and HRQL scores in three domains, emotional, physical, and social, using a 7-point Likert scale with higher scores indicating better HRQL [6]. Construct validity of the 3-factor structure of the MacNew has been substantially confirmed in studies using factor analysis and structural equation modelling [8–12].

Although tools to assess disease-specific HRQL among patients with CAD have been developed, validated and extensively utilized internationally [5, 13], Sri Lanka lacks a validated language-specific tool to assess disease-specific HRQL among patients with CAD. The present study was carried out to assess the construct validity of the interviewer-administered Sinhalese version of the MacNew HRQL instrument among patients being managed for stable CAD.

## Methods

### Participants and procedure

This validation study was conducted in the outpatient clinics of the premier public sector cardiology unit in Sri Lanka that were visited by the principal investigator daily to identify potential study participants. Patients with clinically diagnosed stable angina who were being or had been managed surgically (patients 4–8 weeks prior to and 12–16 weeks following coronary revascularization) or who were being managed medically were recruited for the study. Stable angina was defined as chest pain or discomfort that typically occurs with same amount of activity or emotional stress and is improved or relieved by rest and/or nitroglycerin [14]. The diagnosis of angina was made on the basis of angina symptoms and a positive stress test. The exclusion criteria included critically ill patients, patients with communication barriers (e.g., hearing/speech problems or those who cannot communicate in Sinhala) and patients with any other major illness/condition such as malignancy, residual effects of a cerebrovascular accident, limb amputation, severe obstructive airway disease, psychotic illnesses, alcoholic liver disease which can also affect HRQL.

Sampling was done using quota sampling method with special attention to ensure sufficient variability in clinical and socio-demographic characteristics. The sample size was based on recommendations by Tabachnick and Fidell [15] for confirmatory factor analysis (CFA) where 200 is considered adequate for small to medium sized models and we included equal proportions of patients from the following categories: medically managed, pre-coronary revascularization, and post-coronary revascularization. Consecutive recruitment was carried until the required sample size from each group was reached. The stability of the questionnaire was assessed by test-retest reliability where MacNew was re-administered to a sub-sample of 20 medically managed patients two weeks after baseline. The Ethical Review Committee of the Faculty of Medical Sciences at the University of Sri Jayawardhenepura, Sri Lanka approved the study and informed written consent was obtained prior to data collection. Trained interviewers administered the data collection instruments once informed written consent was obtained.

### Measurements

Three interviewer administered questionnaires (IAQ) were used for data collection during this study;

- IAQ- I: Questionnaire on Basic socio-demographic and clinical characteristics
- IAQ- II: MacNew HRQL questionnaire (MacNew)
- IAQ- III: World Health Organization’s quality of life—brief questionnaire (WHOQOL-BREF)

The variables included under IAQ- I were age, sex, main treatment modality, type of revascularization (PTCA/CABG), surgical status (pre-op/post-op) and grading of angina. Angina grading was done according to the Canadian cardiovascular society grading (CCS grading).

The Sinhalese translation of the MacNew was carried out according to standard forward- and backward-translation process [16, 17]. Two independent bilingual translators (one health professional, one lay person) performed the forward translation. Two different independent bilingual translators (one health professional, one lay person) translated back to English. The back-translated versions were compared with the original English version by the original authors of the MacNew to verify whether the original meaning of the items had been retained. This iterative process was continued until consensus was achieved for each item and answering option with adequate semantic comparability of the translated Sinhalese version to the original English MacNew. The study instrument was then pre-tested and used as an interviewer-administered questionnaire. The original 27-item MacNew yields three domain scores and a Global score and scoring was done according to guidelines of the MacNew collaboration [6].

The validated Sinhalese version of WHOQOL-BREF was used to assess concurrent validity of the Sinhalese MacNew.

### Data Analysis

1. Clinical and socio-demographic characteristics of the participants are described as categorical variables.
2. Construct validity was assessed by factor analysis (FA) and known group comparison. Suitability of data for FA was assessed [18] and found to be satisfactory. Factor analysis was conducted in two steps: exploratory factor analysis (EFA) by using principle component analysis (PCA) with oblimin rotation to identify the underlying factor structure and confirmatory factor analysis (CFA) to confirm the identified factor structure. Confirmatory factor analysis was performed using LISREL 8.8 using the robust maximum likelihood (RML) considering the non-normality of data distribution [19]. The present study used χ^2^/df, root mean square error of approximation (RMSEA) and comparative fit index (CFI) to evaluate model fit. A model is considered good if following criteria are met: χ^2^/df < 2 [15], RMSEA <0.05 and CFI >0.95 [20]. Using known groups comparison, the median Global MacNew scores of a subsample of patients with a) less severe angina (CCS grade < II) versus more severe angina (CCS grade ≥ II) and b) pre- versus post- revascularization were compared using Mann-Whitney U test.
3. Concurrent validity was assessed by calculating Pearson correlation (r) between compatible domain scores of MacNew and WHOQOL-BREF. Results were interpreted as follows: small *r* = 0.10–0.29, medium *r* = 0.30–0.49 or large *r* = 0.50–1.0 [18].
4. Reliability was assessed for the Global and each domain using Cronbach's α for internal consistency and intraclass correlation coefficient (ICC) for test-retest reliability. Cronbach’s α >0.70 and ICC >0.9 were considered as satisfactory [21].

SPSS version 19.0 was used in the data analysis and the level of statistical significance was considered as *p* < 0.05.

## Results

### Patients

The study sample consisted of 200 patients with stable angina; selected socio-demographic and clinical characteristics are given in Table 1. There were more surgically managed (67.0 %) than medically managed patients (33.0 %); angina was present in 56.0 % of the patients with the majority in CCS Grade I and II (76.0 %). Using quota sampling equal proportions of males (50 %, *n* = 100) and females (50 %, *n* = 100) were recruited ensuring an adequate representation of females. The mean age of the study participants was 57.7 years (SD, 8.7) with a range from 38 to 75 years.

**Table 1:** Selected socio-demographic and clinical characteristics of the 200 patients with stable angina

Characteristic	Frequency	%
Sex		
Male	100	50 %
Female	100	50 %
Age (years)		
≤ 40	8	4.0
41–50	31	15.5
51–60	83	41.5
> 60	78	39.0
Treatment Option (*n* = 200)		
Medical	66	33.0
Surgical	134	67.0
Type of Revascularization (*n* = 134)		
CABG	65	48.5
PTCA	69	51.5
Surgical Status (*n* = 134)		
Pre-operative	67	50.0
Post-operative	67	50.0
Anginal Status (*n* = 200)^a^		
Angina present	112	56.0
No Angina	88	44.0
CCS Grading of Angina (*n* = 112)		
Grade I	50	44.7
Grade II	35	31.3
Grade III	20	17.9
Grade IV	7	6.1

*CABG* Coronary Artery Bypass Graft, *PTCA* Percutaneous Transluminal Coronary Angioplasty, *CCS* Canadian Cardiovascular Society

^a^Presence of angina symptoms during previous 2 weeks

### MacNew HRQL scores

All patients completed the questionnaire with no missing items. The mean Sinhalese Global MacNew score was 5.0 (SD, 0.93) with a mean of 5.2 (SD, 1.04) on the *Emotional* domain, 4.9 (SD, 1.02) on the *Physical* domain, and 4.8 (SD, 1.00) on the *Social* domain. The Global and each domain score showed a negatively skewed distribution (Table 2).

**Table 2:** Descriptive statistics [mean and standard deviation (SD), interquartile range (IQR)] of the 27 item Sinhalese MacNew scores

Statistics	MacNew scores			
	Global	Emotional	Physical	Social
Mean (SD)	4.96 (0.93)	5.15 (1.04)	4.86 (1.02)	4.84 (1.00)
Median	5.07	5.36	5.00	4.92
(IQR)	(4.33–5.74)	(4.36–5.93)	(4.08–5.60)	(4.17–5.62)
Skewness (SD)	−0.46 (0.17)	−0.52 (0.17)	−0.39 (0.17)	−0.38 (0.17)
Kurtosis (SD)	−0.41 (0.34)	−0.51 (0.34)	−0.32 (0.34)	−0.49 (0.34)
Minimum | Maximum	2.30 | 6.89	2.21 | 6.79	1.70 | 7.00	2.15 | 7.00

### Construct Validity

#### Exploratory Factor Analysis

Using PCA, the unrotated solution with six components exceeding an eigen value of 1 explained 65.2 % of total variation. Direct oblimin rotation was used on the results of the 6-factor solution considering the medium size correlation (0.4) between Component 1 and 3. A decision was taken to retain the first three components or domains as the majority of the items loaded strongly (>0.4), explaining 51.0 % of the total variance which was consistent with the original factor structure of the Sinhalese MacNew. The pattern matrix revealed two items, #17 (*Sports/exercise limited*) and #27 (*Sexual activities*), with low communality values (<0.3) which did not load on any of the components indicating their lack of alignment with the 3-factor model. Item #27 was eliminated for socio-cultural reasons but item #17 was retained to facilitate international comparisons.

Using oblimin rotation, PCA of the 26 items with the three forced factors increased the total variance explained from 50.9 to 53.4 % with a more interpretable factor structure (Table 3) as follows:

**Table 3:** Pattern and Structure matrices of the three-factor solution of the 26-item Sinhalese MacNew

Item	Pattern Matrix			Structure Matrix		
	Component			Component		
	1	2	3	1	2	3
MAC4	**.899**	−.205	.036	**.811**	.208	.118
MAC7	**.845**	−.118	.061	**.800**	.276	.152
MAC1	**.806**	−.091	−.112	**.750**	.253	−.021
MAC2	**.748**	.098	−.025	**.789**	.432	.092
MAC8	**.743**	−.004	.042	**.746**	.339	.140
MAC5	**.727**	−.206	.203	**.770**	.577	.092
MAC15	**.643**	.295	−.048	**.661**	.160	.261
MAC3	**.608**	.101	−.070	**.645**	.364	.030
MAC20	**.546**	.181	.159	**.649**	.458	.265
MAC10	**.492**	.426	−.342	**.639**	.585	−.199
MAC21	**.473**	.152	.114	**.557**	.387	.204
MAC13	**.453**	.258	−.423	**.514**	.385	−.315
MAC18	**.448**	.356	.110	**.624**	.579	.236
MAC6	**.433**	.360	.018	**.598**	.559	.142
MAC9	.003	**.772**	−.006	.352	**.772**	.137
MAC19	−.176	**.722**	.103	.165	**.661**	.212
MAC16	−.054	**.683**	−.012	.253	**.656**	.106
MAC14	.052	**.639**	.269	.378	**.712**	.394
MAC11	.329	**.480**	−.015	.545	**.626**	.117
MAC23	.398	**.468**	−.106	.596	**.629**	.034
MAC 17	.275	**.451**	.015	.394	**.520**	−.318
MAC22	−.067	.018	**.758**	.042	.128	**.753**
MAC24	.189	.113	**.517**	.309	.294	**.563**
MAC12	.233	.342	**.467**	.451	.534	**.562**
MAC25	.308	.304	**.445**	.504	.525	**.542**
MAC26	.234	.425	**.439**	.484	.511	**.549**

Extraction Method: Principal Component Analysis

Rotation Method: Oblimin with Kaiser Normalization

Major loadings for each item are given in **bold**

Underlined numbers in the Pattern Matrix indicate the domains to which each item was included in the original factor analysis study of MacNew [22]

All *Emotional* domain items loaded at >0.4 on Component 1 and was confirmed as the *Emotional* domain with items #12 (*Social activities*) and #23 (*Burden on others*) having cross-loadings similar to the original validation study [22]. Component 2 with items #9 (*Shortness of breath*), #14 (*Chest pain*), #16 (*Aching legs*), #17 (*Sports/exercise limited*) and #19 (*Dizzy or light-headed*) which concentrate on physical symptoms were confirmed as the *Physical* domain. Social interaction items, #12 (*Social activities*), #13 (*Others have less confidence in you*), #22 (*Overprotective family*), #24 (*Excluded*), #25 (*Unable to socialize*) and #26 (*Physically restricted*) loaded on Component 3 which was confirmed as the *Social* domain.

Analysis of the Structure matrix revealed good discrimination between the components (Table 3), i.e., the lowest factor loading of an item within its own domain is higher than the highest factor loading of a cross-loading item from a different domain. This was true for all the *Emotional, Physical*, and *Social* domain items.

Following PCA, the MacNew consisted of a 3-domain structure with 26 items and items were allocated to the domain with the highest loading (Table 4). Based on the above structure, two models were identified for CFA.

**Table 4:** Item allocation to each domain of the 26 item Sinhalese MacNew

Item	Domain		
	*Emotional*	*Physical*	*Social*
MAC1 - *Frustrated*	×		
MAC2 - *Worthless*	×		
MAC3 - *Confident*	×		
MAC4 - *Down in the dump*	×		
MAC5 - *Relaxed*	×		
MAC6 - *Worn out*	×		
MAC7 - *Happy with personal life*	×		
MAC8 - *Restless*	×		
MAC9 - *Shortness of breath*		×	
MAC10 - *Tearful*	×		
MAC11 - *More dependent*		×	
MAC12 - *Social activities*			×
MAC13 - *Others less confidence in you*	×		
MAC14 - *Chest pain*		×	
MAC15 - *Lack self confidence*	×		
MAC16 - *Aching legs*		×	
MAC17 - *Sports/exercise limited*		×	
MAC18 - *Frightened*	×		
MAC19 - *Dizzy or light-headed*		×	
MAC20 - *Restricted or limited*	×		
MAC21 - *Unsure about exercise*	×		
MAC22 - *Overprotective family*			×
MAC23 - *Burden on others*		×	
MAC24 - *Excluded*			×
MAC25 - *Unable to socialize*			×
MAC26 - *Physically restricted*			×
Total	14	7	5

× represent items compatible with the original factor structure

× represent items showing discrepancies with the original factor structure

#### Confirmatory factor analysis

The two models of the Sinhalese MacNew identified for CFA are detailed below.

##### Model 1

The first model for CFA of the MacNew, Model 1, consisted of the 26 items with the following item allocations for the three domains without cross-loadings.

**Table:** 

*Emotional* domain:	Items # 1, 2, 3, 4, 5, 6, 7, 8, 10, 13, 15, 18, 20, 21
*Physical* domain:	Items # 9, 11, 14, 16, 17, 19, 23
*Social* domain:	Items # 12, 22, 24, 25, 26

##### Model 2

A second model for CFA of the MacNew, Model 2, also consisted of the 26 items and was developed taking into consideration the cross-loadings allowed in the original validation of the MacNew [22]. Cross-loading (>0.4) similar to those in the original validation study was seen in three items (items # 10, 13 and 26) in the present validation study (*Pattern matrix* in Table 3). These three items (given in bold) were allocated to more than one domain resulting in the following item allocation:

**Table:** 

*Emotional* domain:	Items # 1, 2, 3, 4, 5, 6, 7, 8, **10**, **13**, 15, 18, 20, 21
*Physical* domain:	Items # 9, **10**, 11, 14, 16, 17, 19, 23, **26**
*Social* domain:	Items # 12, **13**, 22, 24, 25, **26**

The best fitting model was selected by performing CFA using RML estimation as recommended in LISREL 8.8 guidelines [19] for non-normal data.

##### Model fit statistics

Model fit was evaluated based on χ^2^/df < 2, RMSEA <0.05 and CFI >0.95. Neither of the models showed an adequate fit. In Model 1, the CFI showed a fit value above the standard 0.95. However, the other indicators were below the acceptable range of model fit. Therefore, it was decided to modify both models by adding error co-variances and cross-loadings, taking into consideration the conceptual validity of the model fit improvement suggestions made by LISREL.

##### Model fit statistics after modifications

The best fitting model (modified Model 1) consisted of following item allocation (cross-loaded items are given in bold):

Modified Model 1, with the four cross-loading items, showed the best fit (Table 5). In this model, all three model fit criteria considered in the study showed adequate fit [χ^2^/df = 1.39 (χ^2^ = 289.8; df = 209); RMSEA = 0.044 (90 % CI = 0.031 to 0.056); CFI = 0.99)]. Modified Model 1 was significantly different from Model 2 (*p* < .001, _χ2di_ff = 30.03, _dfdi_ff = 1).

**Table 5:** Summary of model fit statistics for the 3-factor model of Sinhalese MacNew after modifications

Model	Fit indices				
	χ^2^	df	p	CFI	RMSEA
Model 1	289.80	209	0.00018	0.99	0.044
Model 2	319.83	210	<0.00001	0.99	0.051

*χ* ^*2*^ Satorra-Bentler scaled Chi-square test, *CFI* Comparative fit index (>0.9 desired), *RMSEA* Root mean square error of approximation (<0.05 desired)

**Fig. 1:**
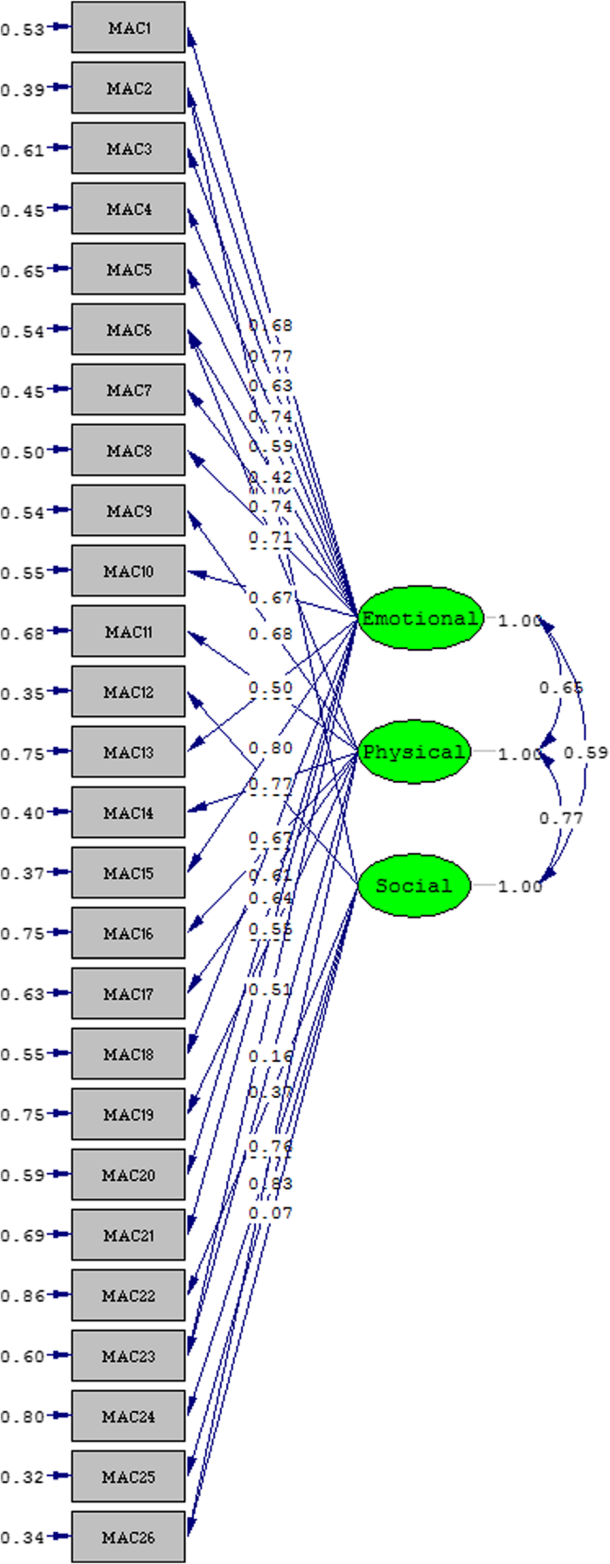
Path diagram of the best fitting model – modified 3- factor 26- item Sinhalese MacNew (Chi-square = 289.80, *P*-value = 0.00018, RMSEA – 0.044)

The factor loadings in CFA indicate how strongly each item is predicted by the underlying latent factors and the standardized parameter estimates of the final modified 3-factor model of the 26-item MacNew are shown in Fig. 1. All the factor loadings of this model are statistically significant at *p* < 0.05.

#### Known group comparison

As an additional approach to establishing construct validity, we compared median Sinhalese MacNew HRQL scores in the sub-groups of patients with selected clinical characteristics, CCS grade and revascularization as an additional method of assessing construct validity using the Mann-Whitney U test (Table 6). Patients with CCS grade I had a significantly lower HRQL (Md = 5.35, *n* = 138) than patients with a CCS grade ≥ II (Md = 5.17, *n* = 62). Post-revascularization HRQL was significantly higher (Md = 5.11, *n* = 67) than pre-vascularization HRQL (Md = 4.78, *n* = 67).

**Table 6:** Comparison of median scores for Global Sinhalese MacNew scores according to angina severity and revascularization status

Clinical category	No:	Median (IQR)	Significance^a^
Angina severity (*n* = 200)			
CCS^b^ grade < II	138	5.35 (4.43–5.84)	U = 1556.5 Z = -2.092 *p* = 0.036
CCS grade ≥ II	62	5.17 (4.34–5.69)	
Revascularization status (*n* = 134)			
Pre-revascularization	67	4.78 (4.11–5.18)	U = 1646.5 Z = 2.78 *p* = 0.005
Post-revascularization	67	5.11 (4.44–5.62)	

*IQR* interquartile range

^a^Mann-Whitney U test

^b^Canadian Cardiovascular Society grading of angina

### Concurrent Validity

Pearson correlations (r) between compatible domain scores of Sinhalese MacNew and WHOQOL-BREF were statistically significant with highest correlation observed in the *Emotional* domain (*r* = 0.79) followed by *Physical* (*r* = 0.73) and *Social* (*r* = 0.36) domains.

### Reliability of The MacNew

#### Internal consistency

Internal consistency of the Sinhalese MacNew was assessed separately for the Global and each domain scales with Cronbach’s α of 0.92 for the *Global* scale and 0.91 for the *Emotional*, 0.85 for the *Physical* and 0.86 for the *Social* domains (Table 7).

**Table 7:** Internal consistency and test-retest reliability of the overall instrument and the three domains of the Sinhalese MacNew

Internal Consistency	No. of items	Cronbach's α
Global	26	0.92
Emotional	14	0.91
Physical	13	0.85
Social	13	0.86
Test-retest reliability	ICC^a^	
Global	0.99	
Emotional	0.99	
Physical	0.99	
Social	0.99	

^a^Intraclass Correlation Coefficient

#### Test-retest reliability

Test-retest reliability of the MacNew was also found be satisfactory with global and all three domain scores having high correlations (ICC > 0.99) (Table 7).

## Discussion

Sri Lanka lacks a language specific validated tool to assess disease-specific HRQL among Sinhalese patients with CAD. The MacNew HRQL questionnaire was therefore translated into Sinhalese and was tested for reliability and validity in 200 patients with stable angina. The original 3-factor model (Physical, Emotional and Social) of the self-administered MacNew with cross-loadings was confirmed with the interviewer-administered Sinhalese MacNew with adequate fit for each of the three model fit criteria considered [RMSEA = 0.044 (90 % CI = 0.031 to 0.056); CFI = 0.99; χ^2^/df = 1.39]. Internal consistency of the Sinhalese MacNew was acceptable with Cronbach's α of 0.92 on the Global score and domain scores ranging from 0.85–0.91. Test-retest reliability was also found to be satisfactory with an ICC of >0.9 for the total and each domain. The interviewer-administered Sinhalese MacNew is a reliable and valid patient-reported outcome measure that can be recommended for assessing disease specific HRQL among Sinhalese patients with stable CAD.

However, since most items loaded strongly onto the first three of six components with EFA using PCA as the extraction method, a forced 3-factor, direct oblimin rotation was applied to the results of the 6-factor solution. As the original MacNew analyses used varimax rotation [6, 22], this may explain the observations in the present study where direct oblimin rotation was used. With PCA, the majority of items in the present forced 3-factor model loaded strongly on only one component, or domain, making the identification of three components straightforward. Since all items, except for item 27 (*Sexual activities*) which was deleted for cultural reasons, loaded on factors in a similar way to the original scoring [22], but with less cross-loading, the three components, *Emotional, Physical* and *Social*, were consistent with the original 3-domain MacNew, resulting in the 26-item Sinhalese version of the MacNew.

Two items in the MacNew failed to load >0.4 on any of the components (item #17, *Sports/exercise limited* and item #27, *Sexual activities*) and both had low communalities. This may be explained by the socio-cultural context of the study population. During administration of the questionnaire, it was evident that respondents gave an answer to item #17 which they considered as suitable even though their disease did not affect their sports/exercise activities as these activities were not considered as part of their daily routine. Although it was felt that this item failed to tap the impact of CAD on HRQL in the Sri Lankan cultural context, a decision was taken to retain item #17 to facilitate international comparisons. With regards to item #27 on “Sexual activities”, it was apparent that respondents were reluctant to genuinely answer the question and that social desirability bias may have played a role in the answers given by the Sri Lankan patients. Since this item had also been omitted from the original and Farsi validation studies [22, 23], a decision was taken to proceed with analysis without item #27 for cultural reasons. However, this problem might not have arisen if the MacNew had been self-administered as originally intended. In other language validation studies MacNew was used as a “self-administered” questionnaire. With the lack of reading comprehension in a large number of the study participants and in the nation, the current study used MacNew as an “interviewer-administered” questionnaire. The effects of questionnaire administration mode on final outcome of HRQL studies is inconclusive: some studies have documented evidence for higher mean values in the interviewer-administered mode compared to self-administered while others have concluded that the mode of administration has no effect on the final outcome [24].

The decision to include all items with a factor weight of >0.40 in the original factor analysis [22] lead to cross-loading of items. Of the 26 items, all but four (items #11, #20, #21 and #23) loaded consistently with the item allocation of the original domain scoring system of the MacNew [22]. Content analysis revealed that the *Physical* domain on to which items #11 *(More dependent)* and #23 *(Burden on others)* had loaded could be justified considering the fact of being more dependent due to impaired physical fitness and therefore feeling like a burden on others. However, items #20 *(Restricted or limited)* and #21 *(Unsure about exercise)* loading on the *Emotional* domain rather than on the *Physical* domain could not be readily explained through the concepts of HRQL. Item #20 cross-loaded at 0.458 onto the *Physical* domain as in the original factor structure [6]. This may well be due to the ambiguous wording of this item: “In general, during the last 2 weeks how much have you been restricted or limited as a result of your heart problem?” Only item #21 loaded on to the *Emotional* component without cross-loading on to either the *Physical* or *Social* domains to which it was originally assigned. The Sinhalese version of the MacNew obviously taps more into the emotional concept of this item, addressing more the feeling of being insecure, than the actual performance of physical exercise. However, a decision was taken to retain item #21 to facilitate international comparisons.

Since some researchers have questioned the validity of including all items with a factor weight of >0.40 [25, 26], item allocation for the Sinhalese MacNew was initially done according to the highest factor weight resulting in a structure without cross-loadings (Model 1). This model (Model 1) explained 53.42 % of the total variance and was then subjected to CFA using MRL estimation compared with another 26-item 3-factor model (Model 2) similar to it except for allowing three cross-loadings. Model 1, with the addition of four cross-loadings as suggested by LISREL demonstrated the best fit of these data. Since the χ^2^ value can be affected by a large sample size, its validity in assessing model fit has been questioned [19] and the ratio between the χ^2^ value and the degrees of freedom (df) has been recommended as a better indicator of model fit than the χ^2^ value alone [15]. The χ^2^/df ratio should be <2 in order to claim adequate fit and, in Model 1, the ratio was 1.39 indicating adequate fit. Although contrasting findings have been reported, for example in the English MacNew [25] and the German MacNew [27], the CFA in the current study suggested a 3-factor model for the MacNew. The interviewer-administered Sinhalese 3-factor MacNew is consistent with the original 3-factor structure of the self-administered MacNew [6] that has been substantially confirmed in each of the independently generated study reports in languages as diverse as Norwegian [11], Dutch [10], Chinese [28], Spanish [9] and Hungarian [29].

Comparison of the median MacNew scores on selected clinical characteristics was performed as an additional method of assessing construct validity. As expected, higher Global HRQL scores were observed in patients with less severe angina (CCS grade I) as opposed to more severe angina (CCS grade ≥ II) and patients in the post-operative period as opposed to pre-operative period. Comparison of MacNew HRQL scores in clinically different groups has yielded results similar to those observed in present study [6, 22, 30, 31].

Concurrent validity of the Sinhalese MacNew was also found to be satisfactory with statistically significant Pearson correlation values ranging from 0.36–0.79. The value for the *Social* domain is lowest at *r* = 0.36 which could be due to the fact that the social items of the WHOQOL-BREF do not match the content of the social items of the MacNew.

In international studies, the reliability of the self-administered MacNew is high with Cronbach's α internal consistency values for the three domains reported to range from 0.82 to 0.97 and ICC values ranging from 0.73–0.95 [6, 8–11, 23, 25–28, 30]. In the present study, the Sinhalese interviewer-administered MacNew demonstrated similar results with both Cronbach's α of 0.92 for Global and 0.91, 0.85 and 0.86 for Emotional, Physical and Social domains respectively and an ICC of >0.99 indicating a satisfactory level of test-retest reliability.

The main limitation of this study is the fact that the Sinhalese MacNew was interviewer-administered and not self-administered. Although there are legitimate reasons for this as a large proportion of both the study and national populations lack reading comprehension, this raises the problem of comparing the present results with other published data where the MacNew was self-administered. However, the reliability and validity results with interviewer administration are largely consistent with the results of the self-administered studies. A second limitation is the lack of results for formal content validity index indicating the content validity of the translated tool. However, this has been addressed by the iterative standard forward- and backward- translation process, which was carried out until all conceptual and cultural translational issues had been resolved with the aid of the translators and authors of the original version. The absence of MacNew responsiveness data is another limitation but, now that the reliability and validity of the Sinhalese MacNew has been established, this can be remedied by focusing on responsiveness as an outcome in future studies conducted in Sri Lanka.

## Conclusions

The psychometric analyses of the Sinhalese MacNew demonstrate good construct validity and reliability in stable angina patients in Sri Lanka. The observations in the present study confirm the multidimensionality of the 26-item interviewer-administered MacNew with 3 domains comparable to the original English version and numerous other language versions. The psychometric analyses of the Sinhalese MacNew demonstrate good construct validity and reliability. The interviewer-administered disease-specific Sinhalese MacNew is a valid and reliable patient-reported outcome measure and can be recommended for assessing HRQL in stable angina patients in Sri Lanka.
